# Semaglutide and Opioid Overdose Risk in Patients With Type 2 Diabetes and Opioid Use Disorder

**DOI:** 10.1001/jamanetworkopen.2024.35247

**Published:** 2024-09-25

**Authors:** William Wang, Nora D. Volkow, QuangQiu Wang, Nathan A. Berger, Pamela B. Davis, David C. Kaelber, Rong Xu

**Affiliations:** 1 Center for Science, Health, and Society, Case Western Reserve University School of Medicine, Cleveland, Ohio; 2 National Institute on Drug Abuse, National Institutes of Health, Bethesda, Maryland; 3 Center for Artificial Intelligence in Drug Discovery, Case Western Reserve University School of Medicine, Cleveland, Ohio; 4 Center for Community Health Integration, Case Western Reserve University School of Medicine, Cleveland, Ohio; 5 Center for Clinical Informatics Research and Education, The MetroHealth System, Cleveland, Ohio; 6 Laboratory of Neuroimaging, National Institute on Alcohol Abuse and Alcoholism, National Institutes of Health, Bethesda, Maryland

## Abstract

This cohort study uses emulation target trial methods to evaluate whether semaglutide is associated with lower rates of opioid overdose among patients with type 2 diabetes (T2D) and opioid use disorder (OUD).

## Introduction

Drug overdose fatalities in the United States remain high, with an estimated 107 543 deaths in 2023, mostly from opioids.^1^ Despite the effectiveness of medications for opioid use disorder (OUD) in preventing overdoses, only an estimated 25% of individuals with OUD receive them,^2^ and close to 50% discontinue treatment within 6 months. There is an urgency for alternative treatments for OUD. Glucagon-like peptide-1 receptor agonists (GLP1-RAs), used for type 2 diabetes (T2D) and obesity, modulated dopamine reward signaling and decreased drug rewards, including heroin in rodents.^3^ Anecdotal reports of reduced drug craving in individuals using semaglutide, a new generation GLP-1RA, along with empirical studies showed its therapeutic benefits in alcohol and nicotine use disorders.^4,5^ This led us to investigate whether semaglutide could protect against overdoses in patients with OUD.

## Methods

In this cohort study, we conducted an emulation target trial to compare the association of semaglutide vs other antidiabetic medications, ie, insulin, metformin, dipeptidyl-peptidase-4 inhibitors (DPP-4is), sodium-glucose cotransporter-2 inhibitors (SGLT2is), sulfonylureas, thiazolidinediones, and other GLP-1RAs, including liraglutide and dulaglutide, with opioid overdose risk in patients with comorbid T2D and OUD. Each component of the target trial was emulated using electronic health records (EHRs) from the TriNetX Analytics Platform, a federated health research network providing access to deidentified EHRs of 116.6 million patients in the US.^6^ Previously, we used TriNetX to study semaglutide’s association with outcomes for alcohol and nicotine use disorders.^4,5^

Eligibility criteria included patients diagnosed with both T2D and OUD; prescribed semaglutide or other antidiabetic medications between December 2017 and June 2023; and with a history of obesity, hypertension, hypercholesterolemia, hyperlipidemia, heart diseases, or stroke. Exclusion criteria were bariatric surgery, pancreatitis, type 1 diabetes, thyroid cancer, or gastroparesis. Patients were classified into semaglutide and other antidiabetes medication groups based on the first prescription during the study period, which was the baseline or index event (eAppendix in Supplement 1). The semaglutide group and each comparison group were separately propensity-score matched for covariates at the baseline to emulate randomization. The main outcome (opioid overdose) and a negative control outcome (medical encounters for congenital malformations, deformations, and chromosomal abnormalities) were examined. Follow-up was from the index event until the outcome, death, loss to follow-up, or 12 months, whichever occurred first. Hazard ratios (HRs) and cumulative incidences were estimated using Cox proportional hazard and Kaplan-Meier survival analyses, with censoring applied. Further details appear in the eAppendix in Supplement 1. We used built-in functions that are implemented on the TriNetX analytics platform using libraries and utilities from R version 4.0.2 (R Project for Statistical Computing), Python version 3.7 (Python Software Foundation), and Java version 11.0.16 (Oracle).

The MetroHealth System, Cleveland, Ohio, institutional review board determined research using TriNetX, in the way described here, is not human subject research, and therefore institutional review board approval was not required and the requirement for informed consent was waived. This study follows STROBE reporting guidelines for cohort studies.

## Results

The study included 33 006 eligible patients: 3034 were prescribed semaglutide (mean [SD] age, 57.4 [11.0] years; 1714 [56.5%] female) and 29 972 were prescribed other antidiabetic medications. Semaglutide was compared with each antidiabetic medication class in patients with comorbid T2D and OUD. Before propensity-score matching, the semaglutide and comparison groups differed by age, sex, ethnicity, and comorbidity conditions, but characteristics were balanced after matching (Table). Semaglutide was associated with a significantly lower risk of opioid overdose during a 1-year follow-up compared with other antidiabetic medications, including other GLP-1RAs, with HRs ranging from 0.32 (95% CI, 0.12-0.89) to 0.58 (95% CI, 0.38-0.87) (Figure). The negative control outcome showed no difference between groups.

**Table.** Semaglutide vs Insulin Groups Before and After Propensity-Score Matching for Baseline Covariates in Patients With Comorbid T2D and OUD

Characteristic	Before propensity-score matching			After propensity-score matching		
	No. of patients (%)		SMD	No. of patients (%)		SMD
	Semaglutide (n = 3034)	Insulin (n = 26 958)		Semaglutide (n = 2790)	Insulin (n = 2790)	
Age at index event, mean (SD), y	57.4 (11.0)	58.9 (12.3)	0.13^a^	57.6 (11.0)	57.5 (11.9)	0.01
Sex						
Female	1714 (56.5)	11 731 (44.1)	0.25^a^	1544 (55.3)	1539 (55.2)	0.004
Male	1171 (38.6)	14 096 (53.0)	0.29^a^	1118 (40.1)	1134 (40.6)	0.01
Unknown	149 (4.9)	771 (2.9)	0.10^a^	128 (4.6)	117 (4.2)	0.02
Ethnicity						
Hispanic/Latinx	204 (6.7)	2055 (7.7)	0.04	193 (6.9)	212 (7.6)	0.03
Not Hispanic/Latinx	2294 (75.6)	18 982 (71.4)	0.09	2102 (75.3)	2091 (74.9)	0.009
Unknown	536 (17.7)	5561 (20.9)	0.08	495 (17.7)	487 (17.5)	0.008
Race						
Asian	22 (0.7)	260 (1.0)	0.03	22 (0.8)	20 (0.7)	0.008
Black	591 (19.5)	6304 (23.7)	0.10^a^	539 (19.3)	575 (20.6)	0.03
White	1911 (63.0)	15 983 (60.1)	0.06	1758 (63.0)	1 729 (62.0)	0.02
Unknown	369 (12.2)	2887 (10.9)	0.04	336 (12.0)	325 (11.6)	0.01
Adverse socioeconomic determinants of health^b^	426 (14.0)	3760 (14.0)	0.002	385 (13.8)	385 (13.8)	<0.001
Problems related to lifestyle^b^	725 (23.9)	5687 (21.4)	0.06	645 (23.1)	647 (23.2)	0.002
Preexisting medical conditions, procedures, and medications						
Obesity^c^	2018 (66.5)	10 955 (41.2)	0.53^a^	1805 (64.7)	1883 (67.5)	0.06
Severe obesity^c^	1626 (53.6)	6578 (24.7)	0.62^a^	1436 (51.5)	1439 (51.6)	0.002
Depression	1906 (62.8)	14 197 (53.4)	0.19^a^	1731 (62.0)	1735 (62.2)	0.003
Mood disorders	2136 (70.4)	16 376 (61.6)	0.19^a^	1944 (69.7)	1939 (69.5)	0.004
Anxiety disorders	2098 (69.1)	15 621 (58.7)	0.22^a^	1906 (68.3)	1907 (68.4)	0.001
Psychotic disorders	208 (6.9)	3052 (11.5)	0.16^a^	197 (7.1)	208 (7.5)	0.02
Behavioral disorders	646 (21.3)	2917 (11.0)	0.28^a^	240 (20.0)	235 (20.9)	0.02
Disorders of adult personality and behavior	225 (7.4)	1 978 (7.4)	0.001	196 (7.0)	185 (6.6)	0.02
Behavioral and emotional disorders with onset usually occurring in childhood and adolescence	277 (9.1)	1461 (5.5)	0.14^a^	240 (8.6)	235 (8.4)	0.006
Chronic pain	2356 (77.7)	16 803 (63.2)	0.32^a^	2145 (76.9)	2183 (78.2)	0.03
Alcohol use disorder	421 (13.9)	6011 (22.6)	0.23^a^	397 (14.2)	386 (13.8)	0.01
Nicotine dependence	1346 (44.4)	14 444 (54.3)	0.20^a^	1240 (44.4)	1233 (44.2)	0.005
Cannabis use disorder	362 (11.9)	3952 (14.9)	0.09	330 (11.8)	327 (11.7)	0.003
Cocaine use disorder	357 (11.8)	4874 (18.3)	0.18^a^	325 (11.6)	298 (10.7)	0.03
Other stimulant disorders	246 (8.1)	2459 (9.2)	0.04	220 (7.9)	190 (6.8)	0.04
Other psychoactive substance related disorders	686 (22.6)	8041 (30.2)	0.17^a^	629 (22.5)	602 (21.6)	0.02
Drug overdose	266 (8.8)	3805 (14.3)	0.17^a^	249 (8.9)	237 (8.5)	0.02
Opioid overdose	101 (3.3)	1443 (5.4)	0.17^a^	96 (3.4)	99 (3.5)	0.006
Substance abuse treatment	77 (2.5)	1109 (4.2)	0.09	76 (2.7)	78 (2.8)	0.004
Methadone	308 (10.2)	3991 (15.0)	0.15^a^	295 (10.6)	293 (10.5)	0.002
Buprenorphine	421 (13.9)	3141 (11.8)	0.06	381 (13.7)	376 (13.5)	0.005
Naltrexone	109 (3.6)	454 (1.7)	0.12^a^	91 (3.3)	91 (3.3)	<0.001
Naloxone	1555 (51.3)	10 090 (37.9)	0.27^a^	1414 (50.7)	1385 (49.6)	0.02
Opioid analgesics	2886 (95.1)	23 728 (89.2)	0.22^a^	2650 (95.0)	2667 (95.6)	0.03
Sedatives/hypnotics	2516 (82.9)	20 233 (76.1)	0.17^a^	2306 (82.7)	2308 (82.7)	0.002
Insulin	1916 (63.2)	12 380 (46.5)	0.34^a^	1730 (62.0)	1755 (62.9)	0.02
Metformin	2313 (76.2)	12 197 (45.9)	0.66^a^	2095 (75.1)	2155 (77.2)	0.05
DPP-4i	574 (18.9)	2581 (9.7)	0.27^a^	505 (18.1)	546 (19.6)	0.04
SGLT2i	721 (23.8)	1149 (4.3)	0.58^a^	574 (20.6)	540 (19.4)	0.03
Sulfonylureas	1004 (33.1)	5566 (20.9)	0.28^a^	902 (32.3)	897 (32.2)	0.004
Thiazolidinediones	237 (7.8)	1152 (4.3)	0.15^a^	211 (7.6)	226 (8.1)	0.02
Other GLP-1RAs						
Any	974 (32.1)	1631 (6.1)	0.70^a^	772 (27.7)	752 (27.0)	0.02
Liraglutide	503 (16.6)	819 (3.1)	0.47^a^	402 (14.4)	390 (14.0)	0.01
Dulaglutide	508 (16.7)	665 (2.5)	0.50^a^	402 (14.4)	368 (13.2)	0.04
Exenatide	168 (5.5)	410 (1.5)	0.22^a^	155 (5.6)	163 (5.8)	0.01
Albiglutide	19 (0.6)	33 (0.1)	0.08	18 (0.6)	16 (0.6)	0.009
Lixisenatide	16 (0.5)	10 (<0.1)	0.09	10 (0.4)	10 (0.4)	<0.001

Abbreviations: DPP-4i, dipeptidyl-peptidase-4 inhibitors; GLP-1RA, glucagon-like peptide 1 receptor agonists; OUD, opioid use disorder; T2D, type 2 diabetes; SGLT2i, sodium-glucose cotransporter-2 inhibitors; SMD, standardized mean difference.

^a^ SMD greater than 0.1, a threshold indicating cohort imbalance.

^b^ Adverse socioeconomic determinants of health (Z55-Z65) include problems related to education and literacy, employment and unemployment, housing and economic circumstances, social environment, upbringing, primary support group including family circumstances, certain psychosocial circumstances, and other psychosocial circumstances. Problems with lifestyle (Z72) included tobacco use, lack of physical exercise, inappropriate diet and eating habits, high-risk sexual behavior, gambling and betting, and other problems related to lifestyle including antisocial behavior and sleep deprivation. For propensity-score matching for adverse socioeconomic determinants of health and problems related to lifestyle, the parent codes (Z55-Z65 and Z72) instead of individual child codes were matched due to the small number for each child code.

^c^ Based on *International Statistical Classification of Diseases and Related Health Problems, Tenth Revision* codes, as listed in the eAppendix in Supplement 1.

**Figure.**
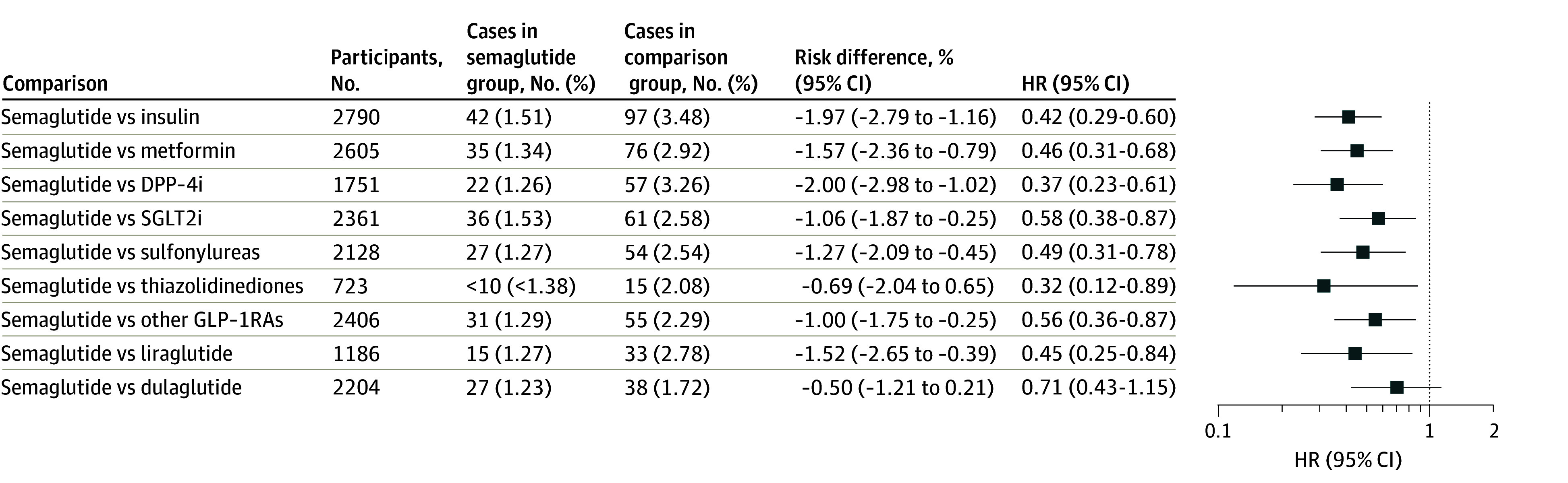
Risk of Opioid Overdose in Patients With Type 2 Diabetes and Opioid Use Disorders, Comparing Propensity-Score Matched Semaglutide With Other Antidiabetic Medication Groups Overall risk was calculated as the number of patients with outcomes during the follow-up time window divided by the number of patients in the cohort at the beginning of the time window. Other glucagon-like peptide 1 receptor agonists (GLP-1RAs) include albiglutide, dulaglutide, exenatide, liraglutide, and lixisenatide. The mean (SD) follow-up times for semaglutide vs each comparison group are as follows: insulin: 342.0 (21.0) days vs 329.0 (32.7) days, metformin: 342.5 (20.6) days vs 337.6 (25.5) days, dipeptidyl-peptidase-4 inhibitors (DPP-4is): 342.1 (20.9) days vs 329.4 (32.1) days, sodium-glucose cotransporter-2 inhibitors (SGLT2is): 341.8 (21.1) days vs 332.1 (29.8) days, sulfonylureas: 341.7 (21.3) days vs 335.6 (27.0) days, thiazolidinediones: 353.5 (10.5) days vs 330.6 (31.3) days, any other GLP-1RA, 342.7 (20.4) days vs 340.6 (22.6) days, liraglutide: 341.6 (21.4) days vs 339.7 (23.5) days, and dulaglutide: 342.6 (20.5) days vs 340.4 (22.7) days. HR indicates hazard ratio.

## Discussion

Semaglutide was associated with reduced opioid overdose risk in patients with comorbid T2D and OUD, suggesting its potential therapeutic value for preventing overdoses. Study limitations include potential unmeasured or uncontrolled confounders, biases, and others inherent in EHR-based observational studies. Results need validation from other data resources and study populations. Further research is warranted to investigate the underlying mechanisms and randomized clinical trials are necessary to corroborate the clinical effects on OUD.

## Notes

Supplement 1. **eAppendix.** Supplementary Methods

Supplement 2.
Data Sharing Statement
