# Supplementary material for: Semaglutide and Opioid Overdose Risk in Patients With Type 2 Diabetes and Opioid Use Disorder
Source: JAMA Netw Open. 2024 Sep 25;7(9):e2435247. doi: 10.1001/jamanetworkopen.2024.35247 (PMC11425147; doi:10.1001/jamanetworkopen.2024.35247)
Supplement: Supplement 1. — eAppendix. Supplementary Methods [file jamanetwopen-e2435247-s001.pdf]

Supplemental Online Content

Wang W, Volkow ND, Wang Q, et al. Semaglutide and opioid overdose risk in patients with type 2 diabetes and opioid use disorder. *JAMA Netw Open*. 2024;7(9):e2435247. doi:10.1001/jamanetworkopen.2024.35247

eAppendix. Supplementary Methods

This supplemental material has been provided by the authors to give readers additional information about their work.

© 2024 Wang W et al. *JAMA Network Open.*

eAppendix. Supplementary Methods

Table of Contents

TriNetX Analytics Platform **.......... 3**

Supplement Figure 1. Graphical illustration of the study design **.......... 4**

Figure 2. Cohort selection flow diagram. **.......... 5**

Supplement Table 1. Specification and emulation of pragmatic target trials. **.......... 6**

Supplement Table 2. Eligibility criteria and exposure definitions. **.......... 9**

Supplement Table 3. Outcome definitions. **.......... 11**

Supplement Table 4: Definitions of covariates. **.......... 11**

© 2024 Wang W et al. *JAMA Network Open.*

TriNetX Analytics Platform

The data used in this study were collected and analyzed on July 20, 2024 within the TriNetX Analytics platform based on the"Research US Collaborative Network". We used the TriNetX platform to access aggregated and de-identified electronic health records (EHRs) of 116.6 million patients from 66 healthcare organizations in the US across 50 states, covering diverse geographic regions, age, race/ethnic, income and insurance groups and clinical setting. TriNetX, LLC is compliant with the Health Insurance Portability and Accountability Act (HIPAA). Any data displayed on the TriNetX Platform in aggregate form, or any patient level data provided in a data set generated by the TriNetX Platform only contains de-identified data as per the de-identification standard defined in Section §164.514(a) of the HIPAA Privacy Rule. TriNetX built-in analytic functions (e.g., incidence, prevalence, outcomes analysis, survival analysis, propensity score matching) allow for patient-level analyses, while only reporting population level data. The MetroHealth System, Cleveland OH, IRB determined research using TriNetX, in the way described here, is not Human Subject Research, and therefore IRB is not required.

TriNetX is a platform that de-identifies and aggregates EHRs data from contributing healthcare systems, most of which are large academic medical institutions with both inpatient and outpatient facilities at multiple locations, across all 50 states in the US. TriNetX Analytics provides web-based and secure access to patient EHR data from hospitals, primary care, and specialty treatment providers, covering diverse geographic locations, age groups, racial and ethnic groups, income levels and insurance types including various commercial insurances, governmental insurance (Medicare and Medicaid), self-pay/uninsured, worker compensation insurance, military/VA insurance among others.

Self-reported sex (female, male), race and ethnicity data in TriNetX comes from the underlying clinical EHR systems of the contributing healthcare systems. TriNetX maps race and ethnicity data from the contributing healthcare systems to the following categories: (1) Race: Asian, American Indian or Alaskan Native, Black or African American, Native Hawaiian or Other, White, Unknown race; and (2) Ethnicity: Hispanic or Latino, Not Hispanic or Latino, Unknown Ethnicity.

TriNetX completes an intensive data preprocessing stage to minimize missing values. TriNetX maps the data to a consistent clinical data model with a consistent semantic meaning so that the data can be queried consistently regardless of the underlying data source. All covariates are either binary, categorical (which expands to a set of binary columns), or continuous but essentially guaranteed to exist. Missing sex values are represented using "Unknown Sex". The missing data for race and ethnicity are presented as "Unknown race" or "Unknown Ethnicity". For other variables including medical conditions, procedures, lab tests and socio-economic determinant health, the value is either present or absent so "missing" is not pertinent.

© 2024 Wang W et al. *JAMA Network Open.*

Supplement Figure 1. Graphical illustration of the study design

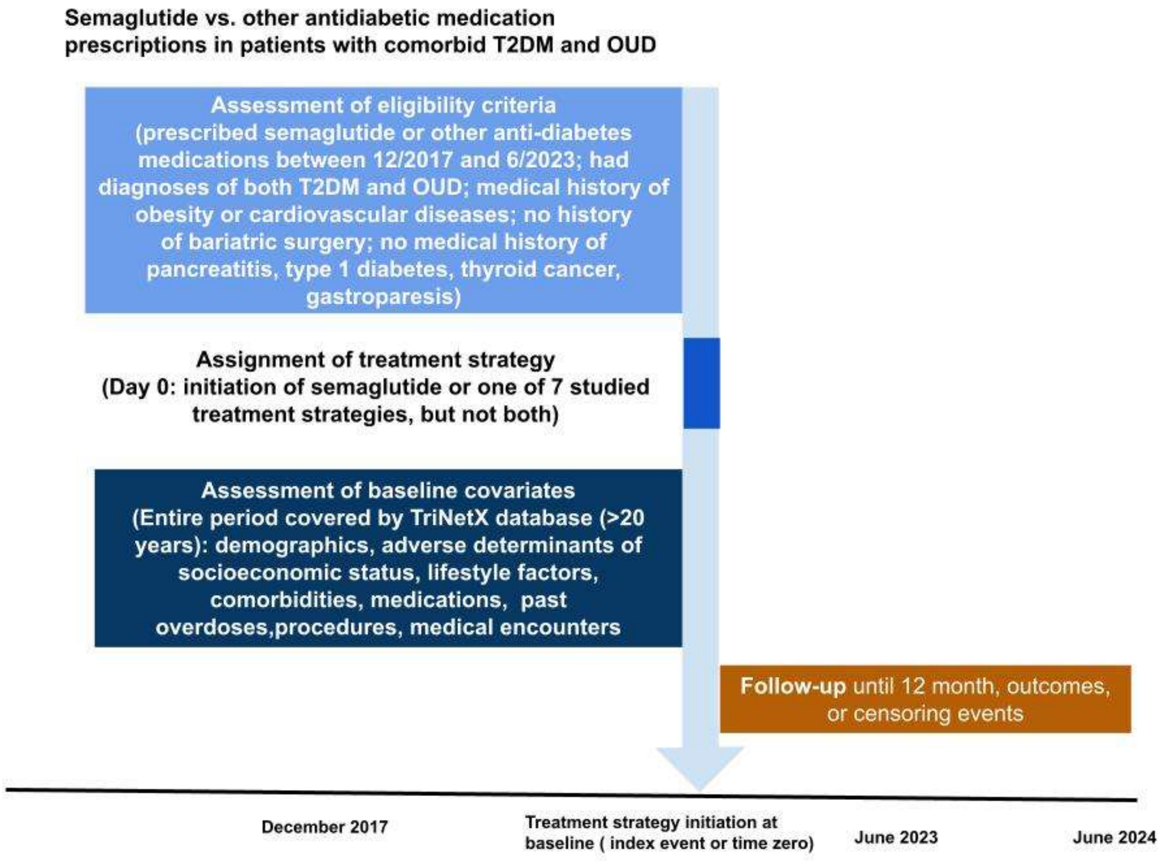

See Supplement Table 2 for definitions of eligibility criteria, exposure, covariates, and outcomes. Follow-up for each individual started at treatment assignment and ended on the day of outcome, death, loss to follow-up, or 12 months after baseline, whichever occurred first. T2DM – Type 2 diabetes; OUD – Opioid use disorder.

© 2024 Wang W et al. *JAMA Network Open.*

Figure 2. Cohort selection flow diagram.

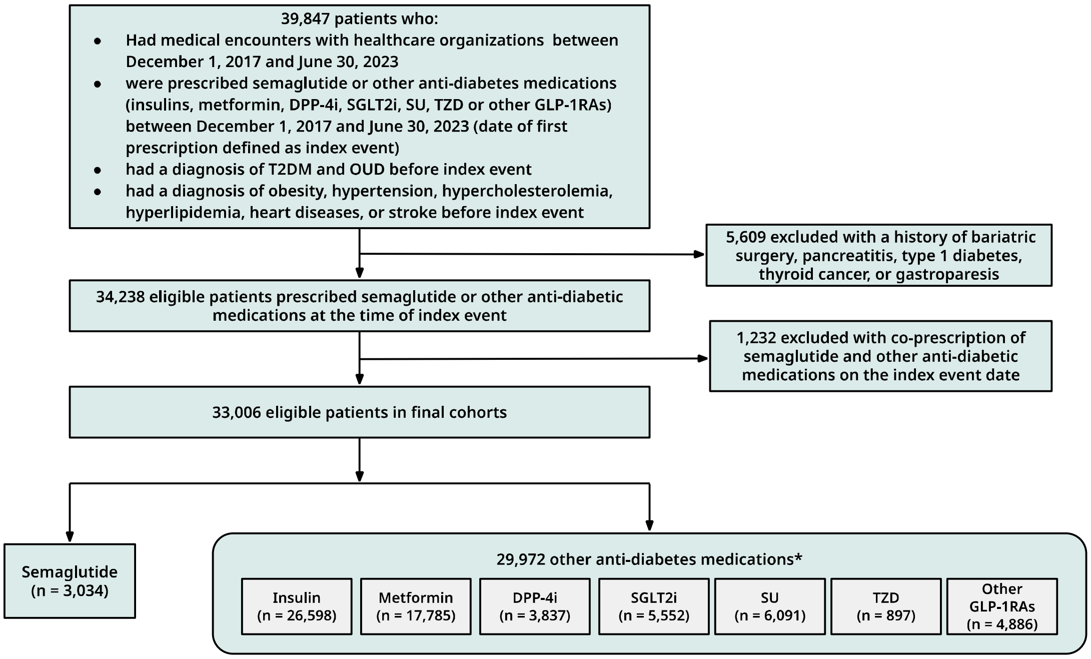

DPP-4i indicates dipeptidyl-peptidase-4 inhibitors; SGLT2i, sodium-glucose cotransporter-2 inhibitors, SU, sulfonylureas, TZD, and thiazolidinediones. Other GLP-1RAs included albiglutide, dulaglutide, exenatide, liraglutide, and lixisenatide. * The combined total of patients (*n* = 29,972) is not a sum of the patients from each of the 7 comparison antidiabetic medication cohorts because a patient could be prescribed more than 1 comparison medications during the study period, though there was no overlap between semaglutide and comparison medications groups.
† Other GLP-1RAs included albiglutide (0.2%), dulaglutide (74.7%), exenatide (6.8%), liraglutide (27.8%), and lixisenatide (1.2%).

© 2024 Wang W et al. *JAMA Network Open.*

Supplement Table 1. Specification and emulation of pragmatic target trials.
Comparing the new use of semaglutide with the new use of other anti-diabetes medications for risk of OUD-related outcomes in patients with comorbid T2DM and OUD using EHR data and analytics functions from the TriNetX Analytics Platform. Target trial specifications and emulations were similar unless otherwise stated.

**Protocol**	**Specification of Target Trials**	**Emulation of Target Trials**
Eligibility criteria	• Prescribed semaglutide or other antidiabetes medications between December 1, 2017 and June 30, 2023<br>• Had a diagnosis of T2DM and a diagnosis of OUD<br>• had at least one of the diseases based on the prescription guideline for semaglutide (obesity, hypertension, hypercholesterolemia, hyperlipidemia, heart diseases, or stroke).<br>• No history of bariatric surgery<br>• No contraindication, warning, and limited use related to semaglutide (pancreatitis, type 1 diabetes, thyroid cancer, and gastroparesis)	Same as for the target trials
Treatment strategies	For the target trial comparing semaglutide vs insulins<br>• Initiate use of semaglutide at index event and not initiate other anti-diabetes medications.<br>• Initiate use of insulins at index event and not initiate semaglutide<br>For the target trial comparing semaglutide vs metformin<br>• Initiate use of semaglutide at index event and not initiate other anti-diabetes medications<br>• Initiate use of metformin at index event and not initiate semaglutide.<br>For the target trial comparing semaglutide vs DPP-4i<br>• Initiate use of semaglutide at index event and not initiate other anti-diabetes medications.<br>• Initiate use of DPP-4i at index event and not initiate semaglutide.<br>For the target trial comparing semaglutide vs SGLT2i	Same as for the target trials. The date of medication initiation was defined as the date of a first medication prescription.

© 2024 Wang W et al. *JAMA Network Open*.

	• Initiate use of semaglutide at index event and not initiate other anti-diabetes medications.<br>• Initiate use of SGLT-2i at index event and not initiate semaglutide.<br>For the target trial comparing semaglutide vs SU<br>• Initiate use of semaglutide at index event and not initiate other anti-diabetes medications.<br>• Initiate use of SU at index event and not initiate semaglutide.<br>For the target trial comparing semaglutide vs TZD<br>• Initiate use of semaglutide at index event and not initiate other anti-diabetes medications.<br>• Initiate use of TZD at index event and not initiate semaglutide.<br>For the target trial comparing semaglutide vs other GLP-1RAs (albiglutide, dulaglutide, exenatide, liraglutide, lixisenatide)<br>• Initiate use of semaglutide at index event and not initiate other anti-diabetes medications.<br>• Initiate use of GLP-1RAs at index event and not semaglutide.<br>For the target trial comparing semaglutide vs liraglutide<br>• Initiate use of semaglutide at index event and not initiate other anti-diabetes medications.<br>• Initiate use of liraglutide at index event and not semaglutide.<br>For the target trial comparing semaglutide vs dulaglutide<br>• Initiate use of semaglutide at index event and not initiate other anti-diabetes medications.<br>• Initiate use of dulaglutide at index event and not semaglutide.<br>•	
**Treatment assignment**	Individuals are randomly assigned to a treatment strategy at baseline. Individuals will be aware of the assigned treatment strategies.	Individuals are assigned to the strategy compatible with their first prescription and assumed randomization by

© 2024 Wang W et al. *JAMA Network Open.*

		propensity-score matching for baseline covariates.
**Outcomes**	• Opioid overdose[1]<br>• Negative control outcome: medical encounters for congenital malformations, deformations and chromosomal abnormalities	Same as for the target trials
**Follow-up**	Follow-up for each individual will start at treatment assignment and end on day of outcome, death, loss to follow-up, or 12 month after baseline, whichever occurs first.	Same as for the target trials
**Casual contrast of interest**	Intention-to-treat: the treatment strategy is assigned at baseline, regardless of medication use adherence, medication switch or add-on.	Observational analog to intention-to-treat
**Statistical analysis**	• Kaplan-Meier estimator to obtain cumulative incidences for each treatment strategy within 12 months of follow-up. Compare cumulative incidence between treatment strategies by risk differences.<br>• Cox proportional hazards analyses to compare rates of time-to-events on daily basis during follow-up time since the baseline.<br>• Models are adjusted for confounders at baseline	Same as for the target trial except observational analogs of intention-to-treat analyses required matching for confounding variables by propensity-score matching.

DPP-4i – Dipeptidyl-peptidase-4 inhibitors; SGLT2i – Sodium-glucose cotransporter-2 inhibitors; SU – Sulfonylureas, TZD – Thiazolidinediones. Other GLP-1RAs include albiglutide, dulaglutide, exenatide, liraglutide, and lixisenatide.

© 2024 Wang W et al. *JAMA Network Open.*

Supplement Table 2. Eligibility criteria and exposure definitions.

**Eligibility criteria**		
**Variable**	**Values**	**Name and Codes**
Diagnosis of T2DM	Binary: present/absent	Type 2 diabetes mellitus (ICD-10 code: E11)
Diagnosis of OUD	Binary: present/absent	Opioid related disorders (ICD-10 code: F11)
Had at least one of the diseases based on the prescription guideline for semaglutide (obesity, hypertension, hypercholesterolemia, hyperlipidemia, heart diseases, stroke).	Binary: present/absent	Hypertension (ICD-10: I10-I1A)<br>Hypercholesterolemia (ICD-10 E78.0)<br>Hyperlipidemia (ICD-10: E78.2, E78.4, E78.5)<br>Heart diseases (ICD-10: I20-I25, I30-I5A)<br>Stroke (ICD-10: I63, I60-I69)<br>Obesity (E66.0, E66.2, E66.8, E66.9, Z68.30, Z68.31, Z68.32, Z68.33, Z68.34, Z68.35, Z68.36, Z68.37, Z68.38, Z68.39, Z68.30, Z68.30, Z68.39, Z68.41, Z68.42, Z68.43, Z68.44, Z68.45)
No history of bariatric surgery	Binary: present/absent	Gastrointestinal System / Bypass / Stomach (ICD-10 Procedure Coding System (PCS): 0D16)<br>Bariatric surgery status (ICD-10: Z98.84)
No contraindication, warning, and limited use where one drug would be preferred over the other (pancreatitis, type 1 diabetes, thyroid cancer, and gastroparesis)	Binary: present/absent	Pancreatitis (ICD-10: K85, K86.0, K86.1)<br>Type 1 diabetes (ICD-10: E10)<br>Gastroparesis (ICD-10: K31.84)<br>Thyroid cancer (ICD-10: C73, Z85.850, E31.2)
**Exposure definitions**		
Initiation of semaglutide at baseline	Binary: present/absent	Semaglutide (RxNorm code: 1991302)
Initiation of insulins at baseline	Binary: present/absent	Insulins (ATC code: A10A)
Initiation of metformin at baseline	Binary: present/absent	Metformin (ATC code: A10BA)
Initiation of DPP-4i at baseline	Binary: present/absent	Dipeptidyl peptidase 4 (DPP-4) inhibitors (ATC code: A10BH)

© 2024 Wang W et al. *JAMA Network Open*.

Initiation of SGLT2i at baseline	Binary: present/absent	Sodium-glucose co-transporter 2 (SGLT2) inhibitors (ATC code: A10BK)
Initiation of SU at baseline	Binary: present/absent	Sulfonylureas (ATC code: A10BB)
Initiation of TZD at baseline	Binary: present/absent	Thiazolidinediones (ATC code: A10BF)
Initiation of other GLP-1RA at baseline	Binary: present/absent	Albiglutide: RxNorm code: 1534763<br>Exenatide: RxNorm code: 60548<br>Dulaglutide: RxNorm code:1551291<br>Liraglutide: RxNorm code: 475968<br>Lixisenatide: RxNorm code: 1440051
Initiation of liraglutide at baseline	Binary: present/absent	Liraglutide: RxNorm code: 475968
Initiation of dulaglutide at baseline	Binary: present/absent	Dulaglutide: RxNorm code:1551291

T2DM – Type 2 diabetes; OUD – Opioid use disorder; DPP-4i – Dipeptidyl-peptidase-4 inhibitors; SGLT2i – Sodium-glucose cotransporter-2 inhibitors; SU – Sulfonylureas, TZD – Thiazolidinediones. Other GLP-1RAs include albiglutide, dulaglutide, exenatide, liraglutide, and lixisenatide. ICD-10 – International Classification of Diseases System, version 10; RxNorm – medical prescription normalized Medical prescription; ATC – Anatomical Therapeutic Chemical (ATC) classification system; CPT – Current Procedural Terminology

© 2024 Wang W et al. *JAMA Network Open.*

Supplement Table 3. Outcome definitions.

**Eligibility criteria**		
**Variable**	**Values**	**Name and Codes**
**Primary outcomes**		
Opioid overdose[1]	Binary: present/absent	Poisoning by opium (ICD-10: T40.0X)<br>Poisoning by heroin (ICD-10: T40.1X)<br>Poisoning by other opioids (ICD-10: T40.2X)<br>Poisoning by methadone (ICD-10: T40.3X)<br>Poisoning by, adverse effect of and underdosing of other synthetic narcotics (ICD-10: T40.4X)<br>Poisoning by fentanyl or fentanyl analogues (ICD-10: T40.41)<br>Poisoning by tramadol (ICD-10: T40.42)<br>Poisoning by other synthetic narcotics (ICD-10: T40.49)<br>Poisoning by unspecified narcotics (ICD-10: T40.60)<br>Poisoning by other narcotics (ICD-10: T40.69)
Negative control outcome		
Congenital malformations, deformations and chromosomal abnormalities	Binary: present/absent	Congenital malformations, deformations and chromosomal abnormalities (ICD-10: Q00-Q99)

ICD-10 – International Classification of Diseases System, version 10
RxNorm – medical prescription normalized Medical prescription
ATC – Anatomical Therapeutic Chemical (ATC) classification system
CPT – Current Procedural Terminology
HL7V3.0 – Health Level Seven (HL7) Vocabulary, Version 3.0

Supplement Table 4. Definitions of covariates.

**Variable**	**Value**	**Code**	**Coding terminology**
Age at Index	continuous	AI	Demographics
Female	Binary: present/absent	F	Demographics
Male	Binary: present/absent	M	Demographics
Black or African American	Binary: present/absent	2054-5	Demographics
White	Binary: present/absent	2106-3	Demographics
Unknown Race	Binary: present/absent	UNK	Demographics
Unknown Gender	Binary: present/absent	UN	Demographics
Not Hispanic or Latino	Binary: present/absent	2186-5	Demographics
Hispanic or Latino	Binary: present/absent	2135-2	Demographics
Asian	Binary: present/absent	2028-9	Demographics
Persons with potential health hazards related to socioeconomic and psychosocial circumstances	Binary: present/absent	Z55-Z65	ICD-10
Problems related to lifestyle	Binary: present/absent	Z72	ICD-10
Schizophrenia, schizotypal, delusional, and other non-mood psychotic disorders	Binary: present/absent	F20-F29	ICD-10
Mood [affective] disorders	Binary: present/absent	F30-F39	ICD-10
Anxiety, dissociative, stress-related, somatoform and other nonpsychotic mental disorders	Binary: present/absent	F40-F48	ICD-10
Behavioral syndromes associated with physiological disturbances and physical factors	Binary: present/absent	F50-F59	ICD-10
Disorders of adult personality and behavior	Binary: present/absent	F60-F69	ICD-10
Alcohol related disorders	Binary: present/absent	F10	ICD-10
Nicotine dependence	Binary: present/absent	F17	ICD-10
Cannabis related disorders	Binary: present/absent	F12	ICD-10

© 2024 Wang W et al. *JAMA Network Open.*

Cocaine related disorders	Binary: present/absent	F14	ICD-10
Other stimulant related disorders	Binary: present/absent	F15	ICD-10
Other psychoactive substance related disorders	Binary: present/absent	F19	ICD-10
Depressive episode	Binary: present/absent	F32	ICD-10
Chronic pain, not elsewhere classified	Binary: present/absent	G89.2	ICD-10
Behavioral and emotional disorders with onset usually occurring in childhood and adolescence	Binary: present/absent	F90-F98	ICD-10
Obesity	Binary: present/absent	E66, Z68.3, Z68.4	ICD-10
Severe obesity	Binary: present/absent	E66.01	ICD-10
Drug overdose	Binary: present/absent	T40	ICD-10
Opioid overdose	Binary: present/absent	T40.0X, T40.1X, T40.2X, T40.3X, T40.4X, T40.41, T40.42, T40.49, T40.60, T40.69	ICD-10
Methadone	Binary: present/absent	6813	RxNorm
Buprenorphine	Binary: present/absent	1819	RxNorm
Naltrexone	Binary: present/absent	7243	RxNorm
Naloxone	Binary: present/absent	7242	RxNorm
Opioid analgesics	Binary: present/absent	CN101	ATC
Sedatives/hypontics	Binary: present/absent	CN300	ATC
Insulins and analogues	Binary: present/absent	A10A	ATC
Biguanides	Binary: present/absent	A10BA	ATC
Sulfonylureas	Binary: present/absent	A10BB	ATC
Thiazolidinediones	Binary: present/absent	A10BG	ATC
Dipeptidyl peptidase 4 (DPP-4) inhibitors	Binary: present/absent	A10BH	ATC
Sodium-glucose co-transporter 2 (SGLT2) inhibitors	Binary: present/absent	A10BK	ATC
Other blood glucose lowering drugs, excl. Insulins	Binary: present/absent	A10BX	ATC
Glucagon-like peptide-1 (GLP-1) analogues	Binary: present/absent	A10BJ	ATC
Liraglutide	Binary: present/absent	475968	RxNorm
Dulaglutide	Binary: present/absent	1551291	RxNorm

© 2024 Wang W et al. *JAMA Network Open.*

Exenatide	Binary: present/absent	60548	RxNorm
Albiglutide	Binary: present/absent	1534763	RxNorm
Lixisenatide	Binary: present/absent	1440051	RxNorm
Substance abuse treatment	Binary: present/absent	H	ICD-10
Hospitalizations	Binary: present/absent	1013659	CPT
Emergency department visit	Binary: present/absent	1013711	CPT

ICD-10 – International Classification of Diseases System, version 10
RxNorm – medical prescription normalized Medical prescription
ATC – Anatomical Therapeutic Chemical (ATC) classification system
CPT – Current Procedural Terminology

© 2024 Wang W et al. *JAMA Network Open.*
